# Occupational burnout and their determinants among schoolteachers in Nepal: a cross-sectional study

**DOI:** 10.1186/s12888-024-05923-9

**Published:** 2024-06-27

**Authors:** Netra Raj Paudel, Prakash KC, Radhika Ghimire, Clas-Håkan Nygård, Subas Neupane

**Affiliations:** 1 Unit of Health Sciences, Faculty of Social Sciences, Tampere University, Tampere, FI-33104 Finland; 2 Health and Population Department, Central Department of Education, Tribhuvan University, Kathmandu, Nepal; 3 Gerontology Research Center, Tampere University, Tampere, Finland; 4 Health System Strengthening Department, RMNCAH Unit, World Health Organization, Kathmandu, Nepal

**Keywords:** Burnout syndrome, Determinants, Schoolteachers, Teaching, Work-related stress

## Abstract

**Background:**

Burnout syndrome attributable to cumulative stressors is highly prevalent among teachers. Despite this, knowledge of burnout syndrome among schoolteachers in lower-middle-income countries are limited, therefore we aimed to investigate self-reported occupational burnout syndrome and associated factors among schoolteachers in Nepal.

**Methods:**

A survey was conducted among randomly selected 37 community schools in Kathmandu, Nepal in 2022, with a total sample of 218 schoolteachers (70% male). Occupational burnout was assessed using the Nepali version of the validated Maslach Burnout Inventory (MBI-ES). MBI-ES consists of 22 items assessing occupational burnout, which were classified into emotional exhaustion (EE, 9 items, score range: 0–45), depersonalization (DP, 5 items, 0–23), and personal accomplishment (PA, 8 items; 3–48). The greater score in EE and DP and the lower score in PA indicate a higher level of burnout. Various socio-demographic, lifestyle, and work-related factors were examined as determinants of occupational burnout using ANOVA and multivariable linear regression models.

**Results:**

The mean scores of EE, DP, and PA were 14.99 (Standard Deviation, SD = 9.79), 4.18 (SD = 4.57), and 42.11 (SD = 6.82) respectively. Poor/moderate work ability contributed to poorer ratings of all three dimensions. Teaching special needs students contributed to EE and DP, whereas low physical activity and alcohol intake were associated with PA only. Younger age, being married, language of teaching, having a disability, sub-optimal physical fitness, poor sleep quality, and ever smoking contributed to EE only.

**Conclusion:**

Occupational burnout among schoolteachers was relatively high. Marital status, lifestyle behavioral, and work-related factors were associated especially with EE and workability was a strong determinant of all three dimensions.

**Clinical trial registration number:**

NCT05626543.

## Introduction

Teachers experience significantly higher stress compared to average population [1] and other professionals [2, 3]. Recent systematic reviews and meta-analyses have highlighted the chronicity of the problem among schoolteachers around the globe with a pooled prevalence of 62.6% [4] with the variation in prevalence between 8.7 and 87.1% [5], which reflects a significant burden of stress among schoolteachers.

Burnout is regarded as a condition that develops because of chronic occupational stress, particularly observed among professionals such as teachers, delivering direct human service [6]. Maslach et al., [7] defined stress as “*an individual’s psychological and physiological response that exceeds their ability to cope or manage effectively”*. When a person has an excessive demand on their individual resources due to high stress, it results in a gradual decline in emotional well-being, loss of motivation, and diminished commitment among workers which is known as “burnout” [8]. Burnout causes a gradual erosion of job engagement resulting from a stressful and frustrating work environment, ultimately reaching a point where it is recognized as a medical condition [7, 9]. Maslach and colleagues developed an instrument for assessing burnout [10–13]. This measurement tool conceptualizes burnout as a three-fold syndrome encompassing emotional exhaustion (EE), depersonalization (DP), and personal accomplishment (PA). Emotional exhaustion refers to experiencing serious fatigue, low energy levels, and a sense of being emotionally drained and depleted. Depersonalization or dehumanization involves adopting negative and cynical attitudes, losing interest and enthusiasm, and lacking empathy toward others. Personal accomplishment entails the ability to tackle new challenges and experience a sense of fulfillment. When absent, it can lead to negative attitudes about one’s job capabilities, decreased effectiveness, reduced work engagement, and affects overall workability. Maslach et al. [7, 9] argued that burnout is a work-related phenomenon, particularly in human service-centered professions. In this study, we used the Maslach Burnout Inventory (MBI) to define occupational stress.

Occupational burnout may have both individual and societal consequences. On an individual level, it may lead to an increase in leave of absence and changing careers or early retirement [14] including poor health conditions [15], which can have long-term negative consequences for society in terms of the quality of education system and economy.

The teaching profession is one of the largest workforces in the world [16] which requires a significant social and emotional demand in everyday work [17]. Teachers are at the center owing to the elevated level of stress due to excessive workload, unhealthy work environment, difficulties in classroom management [2], and role conflict [18]. Likewise, student misbehaviors, parental negative attitudes, unsupportive administrators and colleagues, and lack of adequate technology-friendly infrastructure for learning [19] and additional responsibilities such as administrative tasks, meetings, and record-keeping can add to a teacher’s workload, elevating their stress level [20]. Similarly, personal factors such as sedentary lifestyles and excessive screen time have also been linked to negative physical and mental health outcomes, which may be the other potential contributor to elevated stress levels among teachers [21]. Previous studies have reported that the level of stress among teachers may vary based on their demographic characteristics (age, race, gender, education status, and number of children), and socio-cultural background including quality of life [4, 22].

Knowledge of the occupational well-being of schoolteachers is essential as it is one of the largest occupational groups contributing to the welfare of a country. In addition, the teaching profession comes up with several challenges in lower-middle income countries like Nepal as they are responsible for teaching in large class sizes, working with students with disadvantaged backgrounds, limited resources to perform duties, high workload, and lack of proper opportunities for professional development. Therefore, to fill this knowledge gap, we aimed to assess the prevalence of occupational burnout and the factors contributing to that burnout among secondary-level schoolteachers in Nepal, as a representative population of lower-middle-income countries.

## Materials and methods

This cross-sectional study is based on a survey among community secondary-level schoolteachers of the Kathmandu district in Nepal. The survey was conducted as a baseline measurement for a randomized controlled trial to study the effect of mindfulness-based cognitive behavior therapy on occupational stress management among schoolteachers from July to September 2022.

The study protocol was registered at Clinical Trials (ClinicalTrials.gov ID: NCT05626543) on 22 November 2022, and the ethical committee of the Nepal Health Research Council (225/2022) granted ethical approval to conduct the study. Additional permission was obtained from the Education Development and Coordination Unit of the Kathmandu district and the concerned schools to conduct the study.

### Study population and sample

The study population comprised secondary-level schoolteachers of community schools (government-supported) in the Kathmandu district. We sampled 40 schools out of 165 eligible schools in the Kathmandu district, using a computer-generated random sampling method. It was estimated that there were at least five teachers at the secondary level involved in teaching five different compulsory subjects, which resulted a total of 200 teachers in 40 selected schools.

### Sample size and power calculation

The estimated sample size of 200 participants was proportional to the prevalence of occupational stress among schoolteachers. This study utilized the power of 80%. There was a need for a sample of 90 teachers in each study group and 180 in total and assuming a 10% attrition rate, the participants per study group was 100 and in total 200 teachers. There are a minimum of five teachers in every secondary-level school in Nepal with at least one teacher each for five compulsory subjects. Therefore, we selected 40 schools to reach our target of 200 teachers. The following formulae was used to calculate the sample size.

$$n=\frac{{\text{z}}^{2}p\left(1-p\right)}{{\text{d}}^{2}}$$

Where, n = sample size, Z = Z statistic for a level of confidence (1.96 for 95% CI), p = expected prevalence or proportion, and d = precision.

However, in two targeted schools, none of the teachers provided consent to participate in the study. Similarly, in one school, the teachers agreed to give their consent, but they did not fill up and submit the questionnaire, which reduced the total number of schools to 37. Eligible participants were those working as full-time teachers in the selected schools to the date of the survey with experiences of at least one year. Those teachers who were currently on long-term sick leaves or maternity leave were excluded from the sample selection. Participation in the study survey was voluntary. The written consent was received from the teachers, through a face-to-face formal meeting with them. Detailed study objectives and relevance of the study were explained to each participant in groups before obtaining their consent. They were provided with a consent letter in Nepali language. We successfully obtained consent from 291 teachers and 218 teachers filled up and submitted the online questionnaire sent to them for baseline measurement. Despite the reduction of three schools, the number of required participants was met.

### Assessment tools

The web-based survey was conducted using a sociodemographic questionnaire for assessing socio-demographics, lifestyles, health behaviors, and work-related factors and the Maslach Burnout Inventory- Educators Survey (MBI-ES) tool for measuring occupational stress. The questionnaire was first prepared in English language and translated into the Nepali language, and it took about 25 to 30 min for participants to answer the questionnaire. The socio-demographic questionnaire comprised 32 questions on general demographic information (age, gender, ethnicity, income level, etc.). Lifestyle factors measured were smoking status, alcohol consumption, physical activity, sleep quality, self-rated health, and body mass index which were measured using a previously validated set of questions (Table 1).

**Table 1:** Measurement of variables used in the analysis

Variables name	Original response options	Variable used in the analysis
Age	The year of birth was asked	Two categories were created (< 40 Vs. ≥40)
Gender (Sex)	Male, Female, or option of Other was provided	Two categories were used (Male Vs. Female), none reported ‘other’ gender.
Marital status	Current marital status was asked with following options (single, married, widow, divorced, other)	Two categories were created (married Vs. all others)
Education	Highest degree of education was asked with the following response options (Bachelor, Master, MPhil, doctorate (Ph.D.))	Three categories were created (Bachelor’s, Master’s, MPhil/Ph.D.)
Income/month (NRs)	Total monthly household income was asked in rupees with following options (below 40,000, 41,000–51,000, 51000-60,000, above 60,000)	Two categories were created ≤ 51,000 Rs (Close to 350 Euro) Vs. >51,000 Rs
Teaching language	Medium of language used in teaching was asked with the following options (Nepali, English, Sanskrit, Nepali & English both, other)	Three categories were created (Nepali, English, all others)
Employment status	Employment status was asked by a question with ten possible alternatives.	Two categories of permanent and temporary were created.
Self-rated health	Self-rated health was asked by a question with five possible alternatives (very good, good, average, poor, and very poor).	Two categories were created good (very good and good) and sub-optimal (average, poor and very poor).
Any disability	Any disability was asked in yes/no question.	No change was brought.
Work ability	Work ability was asked in 1 to 10 scales: from poor to excellent.	Categorized into three: poor (0–6), moderate (7–8) and very good (9–10).
BMI	Two questions - height in inch and weight in kg.	Inch changed into meter and created BMI in the two categories, less than 25 kg/m^2^ and 25 or above.
Leisure time activity	Leisure time activity was asked by a question with nine possible alternatives (TV, social media, social work, social gathering, meeting with friends, kitchen, garden, farm, and others).	Four categories were created: sedentary (TV and social media), Social gathering (social work, social gathering, and meeting friends), time spent with family, and kitchen /garden /others.
Physical fitness	Physical fitness was asked in a scale of 1–5 (very good, good, average, poor and very poor).	Modified into two categories, good (very good and good) Vs. sub-optimal (average, poor and very poor).
Physical exercise	Two questions were asked about time and intensity of physical exercise.	A variable with 3 categories were created with low, moderate, and high.
Sleep Quality	A question on the quality of sleep was asked with four alternatives (very good, good, average and poor).	Two categories were created good (very good and good) vs. poor (average and poor).
Smoking	Smoking habit was asked by a question with possible alternatives (yes, never and former using)	Two categories were created - Ever (yes and former), and never.
Chewing tobacco	Use of chewing tobacco was asked a question with possible alternatives (yes, never, and former chewing)	Two categories were created ever (former and yes) vs. never.
Alcohol intake	Alcohol intake was asked by a question with possible three alternatives (yes, never, and former drinking)	Two categories were created ever (former and yes) vs. never.
Comorbidity	Six questions with yes/no options were asked about the presence of heart diseases, cancer, diabetes, asthma, arthritis, and high blood pressure.	Two categories no diseases vs. one or more diseases were created based on six questions.
Teaching hours/day	Teaching period (40 min) per day was asked by a question with possible alternatives: (4 period or less/day, period /day, 5 period/day, 6 period/day and 7 or above).	Three categories were created with ≤ 4, 5 or ≥ 6 h
Class size	Class size was asked by a question with open scale on how many students do you teach in one class.	Two categories, normal (≤ 40) vs. overload (> 40) were created.
Employment Years	Total number of years taught in secondary level was asked in open scale.	Three categories were created: <5 years, 5–20 years, and 21 years or above.
Teaching special needs students	Question was asked if you teach special needs students with yes/no.	No change was made.

### MBI-ES tool

MBI-ES tool is a valid and widely used tool to assess occupational burnout among teachers [23]. The MBI-ES tool consisted of 22 items, measuring three dimensions of occupational burnout: emotional exhaustion (EE), depersonalization (DP), and a low sense of personal accomplishment (PA). Respondents were asked to rate their feelings on a 7-point Likert scale (never (0) to every day (6)), and overall scores were obtained by summing the responses using a specific key for each dimension. The threshold for high burnout levels in each dimension, as established by the MBI-ES producer, is determined by a specific point score [24]. The EE consists of 9 items (Cronbach’s α 0.82), and the DP was constructed from 5 items (Cronbach’s α 0.60), and these subscales exhibit that the greater the value the higher the burnout they imply however the PA which consists of 8 items (Cronbach’s α 0.79) indicates that the greater the score the lower the burnout.

### Tool validation

The questionnaire was then translated into Nepali language and back translation by language experts (BA) to ensure the linguistic validity of the questionnaire. To evaluate and validate the questionnaire, a pilot test was conducted among 20 teachers from four secondary schools with similar settings from the neighboring district Lalitpur. The content of the questionnaire was checked by two research experts.

The Maslach Burnout Inventory-Educators Survey (MBI-ES) was available in the English language. Permission to use the MBI-ES tool for this study was obtained from Mind Garden (MBI-ES Copyright ©1986 by Christina Maslach, Susan E. Jackson & Richard L. Schwab, California, USA) together with a license and translation agreement. The linguistic validity of the Nepali version of the MBI-ES tool was done with the help of reviews by a panel of four experts (two senior researchers (PKC, SN) with expertise in the content as well as the language, one (NP) researcher and teacher with knowledge in the content as well as the language and one language expert (BA).

### Statistical analysis

After the data collection, each questionnaire was thoroughly checked for completeness, and a unique code was assigned to each respondent. The internal consistency and reliability of each domain of MBI-ES questions were tested first. We reported Cronbach’s alpha value as a measure of the internal consistency of the items. We used descriptive statistics to present the prevalence of teacher’s burnout and its subscales. Categorical variables were expressed as frequencies and percentages, while continuous variables were reported as mean ± standard deviation (SD).

Then ANOVA test was used to measure the mean difference in the distribution of all three MBI-ES subscales by socio-demographic, general health, lifestyle, and work-related characteristics. F-value was calculated to report the variation in means of the MBI-ES subscale between and within the sample. The higher F-value in an ANOVA indicates higher variation between sample means relative to the variation within the sample with the lower p-value. The association of age with three MBI-ES sub-scales was presented in scatter plots. For the scatter plots age was used as a continuous variable. A regression line was fitted with the confidence interval bands to show the relationship between age and MBI-ES sub-scales.

Linear regression analysis was used to investigate the relationship between three subscales of MBI-ES and socio-demographic, general health, lifestyle, and work-related characteristics. Beta coefficients along with standard errors are reported from the multivariable models.

All statistical analyzes were performed using IBM’s Statistical Packages for the Social Sciences (SPSS) software, version 29.0.

## Results

### MBI scores

The results in Table 2 exhibit that EE, DP, and PA had an average mean score of 14.99 (SD = 9.79), 4.18 (SD = 4.57), and 42.11 (SD = 6.82) respectively among the studied sample. The categorized MBI-ES shows that 8% of teachers had high EE, while 23% had moderate. Similarly, about 8% of schoolteachers had high DP and 27% had moderate and 11% had high PA and 17% had a moderate level of PA (Table 3).

**Table 2:** Occupational burnout among schoolteachers measured by Maslach burnout inventory (MBI-ES).

	Score range	Cronbach alpha	Mean	SD
Emotional Exhaustion (EE)	0–45	0.82	14.99	9.79
Depersonalization (DP)	0–23	0.60	4.18	4.57
Personal accomplishment (PA)	3–48	0.79	42.11	6.82

**Table 3:** Percentages of experienced burnout on the MBI-ES Subscale

Level of stress	Emotional Exhaustion	Depersonalization	Personal Accomplishment
Low	68.8	65.1	72.5
Moderate	22.9	27.1	16.5
High	8.3	7.8	11.0

**Fig. 1:**
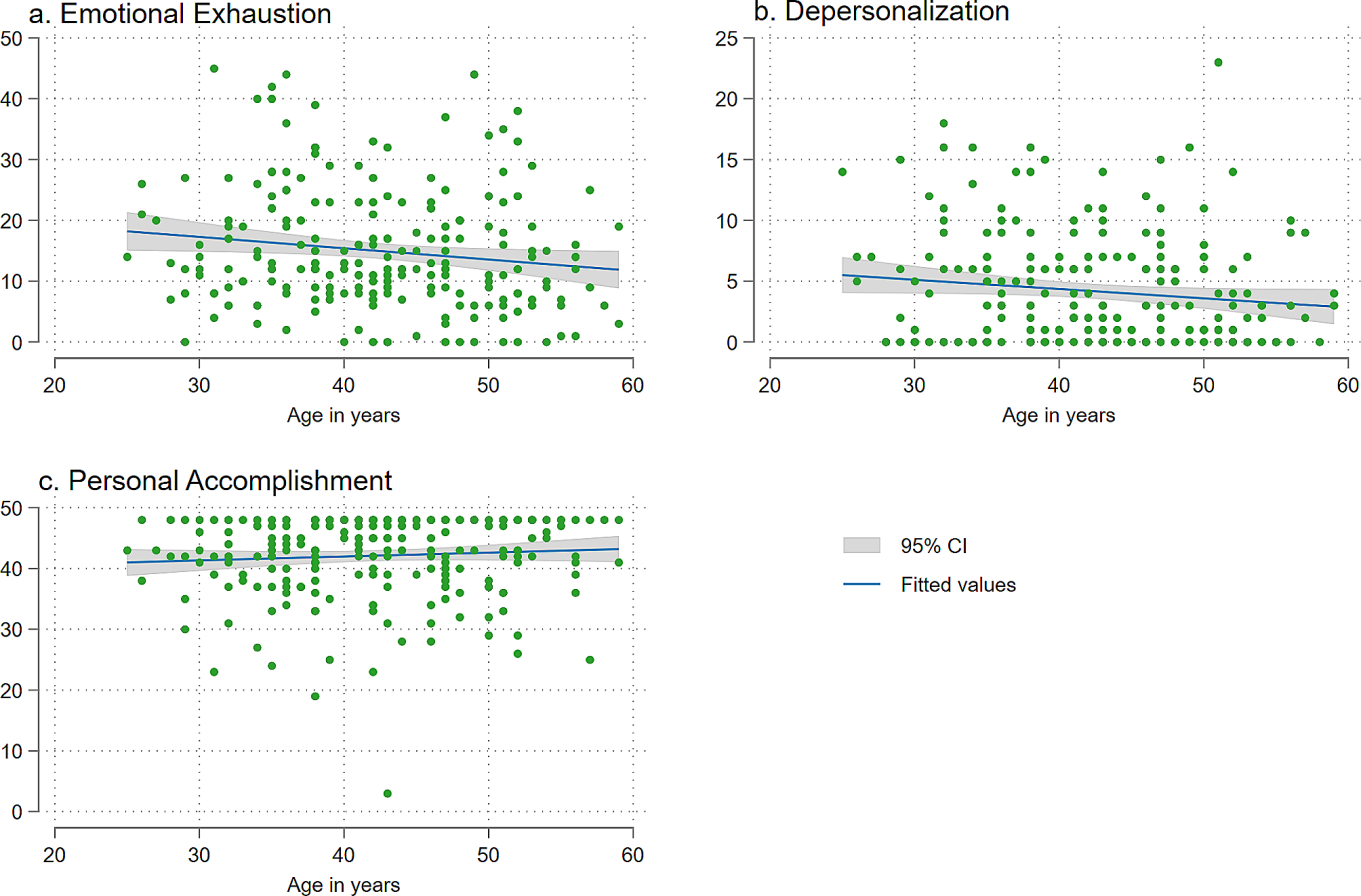
Distribution of teachers’ burnout by age

The distribution of three components of MBI-ES by the age of teachers is presented in Fig. 1. The regression line in Fig. 1(a) shows a slightly decreasing mean level of EE by age with narrow confidence intervals in the middle age around 40 to 50 years. Similar patterns were found for DP. However, the PA was slightly increasing with increasing age.

### Socio-demographic variables and MBI

The distribution of socio-demographic variables is presented by three sub-scales of MBI-ES in Table 4. The ANOVA test was used to explore the variation between the categories of independent variables. EE was significantly higher among younger age teachers (mean 17.34, SD10.41), while elder teachers (≥ 40 years) had 13.49, SD 9.10 (F = 8.30). No significant difference was found between the age groups for DP and PA. Likewise, we did not find any significant difference in EE and DP based on other studied socio-demographic variables, however, a significant difference was found based on gender in PA with females having lower PA than their male counterparts (mean 5.04 vs. 7.33).

**Table 4:** Basic characteristics of the studied population and ANOVA of the MBI subscales by demographic characteristics of the schoolteachers

Characteristics	*N* = 218	Emotional exhaustion			Depersonalization			Personal accomplishment		
		M	SD	F	M	SD	F	M	SD	F
Age										
< 40 years	85	17.34	10.41	8.30 **	4.91	5.03	3.56	41.19	6.49	2.54
≥ 40 years	133	13.49	9.10		3.71	4.22		42.69	6.97	
Gender										
Female	72	15.71	9.99	0.58	3.97	4.28	0.22	44.25	5.04	11.14 ***
Male	146	14.64	9.71		4.28	4.73		41.05	7.33	
Marital status										
Married	198	14.97	9.80	0.006	4.11	4.43	0.55	42.06	6.95	0.12
All others	20	15.15	9.91		4.90	5.90		42.60	5.46	
Education										
Bachelor’s	20	12.20	8.96	0.91	3.85	5.83	0.30	42.55	6.27	0.09
Master’s	188	15.30	9.90		4.16	4.45		42.03	6.97	
MPhil or PhD	10	14.70	9.27		5.20	4.64		42.70	5.12	
Income/month (NRs)										
≤ 50,000	96	15.26	9.76	0.13	4.52	5.03	0.96	41.09	7.66	3.83
> 50,000	122	14.78	9.85		3.91	4.19		42.90	5.98	
Teaching language										
Nepali	89	14.84	8.79	1.86	3.85	4.44	1.86	41.80	7.72	0.75
English	44	17.39	11.71		5.36	5.26		41.41	7.35	
Both (Nep + Eng)	85	13.91	9.60		3.91	4.30		42.79	5.39	
Employment status										
Permanent	129	15.05	10.04	0.01	4.09	4.29	0.11	41.53	7.35	2.23
Temporary	89	14.90	9.48		4.30	4.98		42.93	5.90	

### Lifestyle, work-related factors, and MBI

A significant difference in EE was found for self-rated health, disability, work ability, physical fitness, sleep quality, and smoking (Table 5). Significantly higher EE was found among teachers with sub-optimal health, having a disability, sub-optimal physical fitness, poor sleep quality, and ever smoking. In DP, a significant difference was found in physical exercise, alcohol intake, and teaching special needs students. Teachers with moderate levels of physical exercise, ever drinking alcohol, and teaching special needs students had higher DP. PA level was significantly different based on different categories of work ability, physical exercise, smoking, and alcohol intake. Teachers with very good work ability, high levels of physical exercise, never smoking and never alcohol intake had significantly higher PA.

**Table 5:** General health, lifestyle, and work-related characteristics and burnout of the studied participants

Characteristics	*N*	Emotional exhaustion			Depersonalization			Personal accomplishment		
		M	SD	F	M	SD	F	M	SD	F
Self-rated health										
Good	146	13.12	8.85	17.29***	4.07	4.33	0.26	42.40	7.04	0.81
Sub-optimal	72	18.78	10.55		4.40	5.07		41.51	6.34	
Any disability										
No	196	14.52	9.79	4.66 *	4.10	4.51	0.55	42.40	6.65	3.62
Yes	22	19.23	8.92		4.86	5.22		39.50	7.82	
Work ability										
Poor (0–6)	67	18.33	10.93	8.10 ***	5.24	4.96	3.83 *	39.69	7.00	10.43 ***
Moderate (7–8)	77	15.06	9.10		4.26	4.56		41.74	7.35	
Very good (9–10)	74	11.89	8.51		3.14	4.04		44.68	5.04	
11. BMI										
< 25.0 kg/m^2^	119	14.16	9.40	1.90	4.01	4.54	0.36	41.76	7.39	0.65
≥ 25 kg/m^2^	99	15.99	10.20		4.38	4.64		42.52	6.06	
Leisure time activity										
Sedentary	47	16.34	10.01	0.88	5.36	5.01	1.39	39.66	9.09	2.69 *
Spend with family	82	15.06	9.76		3.90	4.42		42.82	5.60	
Social gathering	45	15.33	9.74		3.64	4.57		42.44	6.21	
Kitchen/garden /Others	44	13.07	9.70		3.98	4.33		43.05	6.25	
Physical fitness										
Good	146	13.12	8.85	17.29 ***	4.07	4.33	0.26	42.40	7.04	0.81
Sub-optimal	72	18.78	10.55		4.40	5.07		41.51	6.34	
Physical exercise										
Low (0.00)	36	14.53	9.93	1.21	4.47	4.12	5.20 **	41.94	7.20	4.08 *
Moderate (low)	75	16.40	10.44		5.33	5.45		40.44	7.74	
High (Moderate)	107	14.16	9.31		3.21	3.82		43.33	5.71	
Sleep quality										
Good	156	13.45	9.48	14.45 ***	4.08	4.55	0.27	42.25	7.08	0.25
Poor	62	18.87	9.57		4.44	4.67		41.74	6.14	
Smoking										
Never	191	14.45	9.16	4.78 *	3.97	4.28	3.29	42.47	6.76	4.51 *
Ever	27	18.81	13.03		5.67	6.21		39.52	6.76	
Chewing tobacco										
Never	197	14.94	9.60	0.05	4.23	4.49	0.24	42.30	6.74	1.66
Ever	21	15.43	11.69		3.71	5.43		40.29	7.40	
Alcohol intake										
Never	188	14.87	9.44	0.22	3.87	4.40	6.50 *	42.47	6.71	4.04 *
Ever	30	15.77	11.91		6.13	5.24		39.80	7.14	
Comorbidity										
No	163	14.66	9.55	0.73	4.17	4.46	0.002	41.89	7.16	0.65
one or more	55	15.96	10.50		4.20	4.96		42.75	5.67	
Teaching hours/day										
≤ 4	73	13.01	8.30	2.27	3.82	4.05	1.69	41.64	7.55	0.26
5	114	16.01	10.59		4.68	5.06		42.29	6.63	
≥ 6	31	15.90	9.58		3.16	3.63		42.52	5.73	
Class size										
Normal	56	15.00	9.11	0.00	3.93	4.25	0.23	41.30	7.41	1.04
Over load	162	14.99	10.04		4.27	4.70		42.38	6.60	
Employment Years										
< 5	27	17.74	12.06	3.58 *	5.04	5.51	1.23	42.44	5.00	0.57
5–20	145	15.46	9.39		4.28	4.58		42.34	7.01	
≥ 21	46	11.91	9.01		3.37	3.90		41.15	7.15	
Teaching special need students										
Yes	34	13.09	6.64	1.53	2.56	3.51	5.14 *	42.50	6.32	0.13
No	184	15.34	10.24		4.48	4.70		42.03	6.92	

### Regression-based associations

A multivariable model for the association of demographic, lifestyle, health, and work-related variables with three subscales of MBI-ES based on linear regression is presented in Table 6. Most of the studied variables were significantly associated with EE. When adjusted for the effect of each other variables in the model, compared to the older age group, younger age teachers had significantly higher EE (β = 0.17, SE = 0.05). Married teachers compared to others had 0.31-unit increased EE and having a master’s degree education compared to MPhil or PhD had 0.20 units of increased EE. Similarly, teachers teaching either in Nepali or English language compared to those teaching using both had significantly higher EE. Teachers having any disability, poor work ability, involved in social gatherings during leisure, non-tobacco chewers, and those with shorter tenure (≤ 20 years) had significantly higher EE. Likewise, having good physical fitness compared to sub-optimal, low physical exercise, good sleep quality, never smoking compared to ever smoking, teaching hours of four or fewer hours per day, and teaching special needs students were significantly associated with lower EE among teachers. Similarly, younger age compared to older, having lower education, lower income, good self-rated health, poor or moderate work ability, low or moderate physical exercise, never having chewing tobacco, and shorter teaching experiences were significantly associated with higher DP among teachers. The magnitude of the estimate was high for poor work ability compared to very good (β = 0.58, SE = 0.10), never having chewing tobacco compared to ever (β = 0.56, SE = 0.17), and for moderate physical exercise compared to high (β = 0.43, SE = 0.08). While normal weight compared to overweight or obese, never smoking compared to ever smokers, never alcohol consumption, teaching in normal class size, and teaching special needs students were significantly associated with lower DP among teachers. The magnitude of the association was strong for teaching special needs students (β=-0.62, SE = 0.12), and never drinking alcohol (β=-0.53, SE = 0.11). For PA as an outcome, none of the studied variables were significantly associated.

**Table 6:** Association of demographic, lifestyle, health, and work-related variables with emotional exhaustion (EE), depersonalization (DP), and personal accomplishment (PA).

	β (SE)		
	EE	DP	PA
Age			
< 40 years	0.17 (0.05)	0.16 (0.09)	-0.05 (0.03)
≥ 40 years	Ref	Ref	Ref
Gender			
Male	0.00 (0.05)	0.09 (0.09)	0.05 (0.03)
Female	Ref	Ref	Ref
Marital status			
Married	0.31 (0.07)	0.08 (0.13)	-0.05 (0.04)
All others	Ref	Ref	Ref
Education			
Bachelor’s	-0.03 (0.12)	0.28 (0.21)	-0.05 (0.07)
Master’s	0.20 (0.09)	0.17 (0.16)	-0.06 (0.05)
MPhil or PhD	Ref	Ref	Ref
Income/month			
≤ 50,000	-0.05 (0.04)	0.14 (0.07)	-0.02 (0.02)
>50,000	Ref	Ref	Ref
Teaching language			
Nepali	0.15 (0.04)	-0.27 (0.09)	-0.06 (0.03)
English	0.22 (0.05)	0.10 (0.10)	-0.02 (0.03)
Both	Ref	Ref	Ref
Employment status			
Permanent	− 0.09(0.04)	-0.05 (0.07)	-0.04 (0.02)
Temporary	Ref	Ref	Ref
Self-rated health			
Good	-0.03 (0.05)	0.14 (0.10)	0.06 (0.03)
Sub-optimal	Ref	Ref	Ref
Any disability			
No	0.18 (0.06)	0.08 (0.12)	-0.05 (0.04)
Yes	Ref	Ref	Ref
Work ability			
Poor (0–6)	0.34 (0.05)	0.58 (0.10)	-0.09 (0.03)
Moderate (7–8)	0.13 (0.05)	0.25 (0.09)	-0.06 (0.03)
Very good (9–10)	Ref	Ref	Ref
BMI			
< 25.0 kg/m^2^	-0.07 (0.04)	-0.13 (0.07)	-0.02 (0.02)
≥ 25 kg/m^2^	Ref	Ref	Ref
Leisure time activity			
Sedentary	0.11 (0.06)	-03 (0.11)	-0.04 (0.04)
Spend with family	0.11 (0.05)	-0.009 (0.10)	0.006 (0.03)
Social gathering	0.17 (0.06)	-0.29 (0.12)	0.02 (0.03)
Kitchen/garden/others	Ref	Ref	Ref
Physical fitness			
Good	-0.21 (0.05)	0.05 (0.10)	-0.04 (0.03)
Sub-optimal	Ref	Ref	Ref
Physical exercise			
Low	-0.18 (0.05)	0.34 (0.10)	-0.008 (0.03)
Moderate	-0.09 (0.04)	0.43 (0.08)	-0.04 (0.03)
High	Ref	Ref	Ref
Sleep quality			
Good	-0.22 (0.04)	0.04 (0.09)	-0.03 (0.03)
Poor	Ref	Ref	Ref
Smoking			
Never	-0.42 (0.08)	-0.37 (0.14)	0.05 (0.05)
Ever	Ref	Ref	Ref
Chewing tobacco			
Never	0.34 (0.09)	0.56 (0.17)	-0.04 (0.05)
Ever	Ref	Ref	Ref
Alcohol intake			
Never	0.04 (0.06)	-0.53 (0.11)	0.03 (0.04)
Ever	Ref	Ref	Ref
Comorbidity			
No	-0.004 (0.05)	0.05 (0.09)	-0.04 (0.03)
one or more	Ref	Ref	Ref
Teaching hours/day			
≤ 4	-0.23 (0.06)	0.29 (0.13)	0.003 (0.04)
5	-0.06 (0.06)	0.33 (0.12)	0.01 (0.03)
≥ 6	Ref	Ref	Ref
Class size			
Normal	-0.02 (0.04)	-0.16 (0.08)	-0.007 (0.03)
Overload	Ref	Ref	Ref
Employment Years			
< 5	0.36 (0.08)	0.15 (0.15)	0.02 (0.05)
5–20	0.21 (0.06)	0.29 (0.11)	0.03 (0.03)
≥ 21	Ref	Ref	Ref
Teaching special needs students			
Yes	-0.16 (0.05)	-0.62 (0.12)	0.02 (0.03)
No	Ref	Ref	Ref

## Discussion

In this cross-sectional study of schoolteachers in Nepal, we aimed to investigate self-reported occupational burnout syndrome and associated factors. The mean level of EE was 14.99, DP 4.18 and PA was 42.11. The categorical sub-scale show that the prevalence of moderate to high EE was about 31%, DP was 35%, and PA was 27%. Factors such as age, gender, poor health conditions, lack of physical fitness, disability conditions, sedentary behaviours, limited work experience, teaching students with special needs, smoking, alcohol consumption, and poor sleep quality were among the other contributors especially for EE.

Many previous studies conducted in low- and middle-income countries used different tools to measure teachers’ stress, which limited direct comparison of our findings with previous studies. Nevertheless, one earlier study from India among secondary-level teachers found a lower level of burnout in two subscales EE and DP of MBI-ES: the mean level of EE, DP were 18.05, and 6.80 respectively, and a higher level of PA (mean 35.75) compared to ours [ 24]. Another study found higher levels of all MBI-ES subscales with EE (mean 19.36), DP (mean 6.14), and PA (mean 36.90) among university teachers in the USA [25].

When using the categorical scale, our findings show a relatively low level of occupational burnout (about one-third) compared to previous studies conducted among schoolteachers in lower-middle and low-income countries like Nigeria (36%) and Ethiopia (37.4%) [26, 27] and upper-middle and high-income countries like China (53%) and Portugal (40%) [28, 29]. However, the prevalence was relatively higher compared to those reported by previous studies among schoolteachers in other countries with different income settings like India 23%, Taiwan (26%), Tunisia (21%), Sweden (15%), Finland (13%), and Srilanka (12%), Brazil (12%) [23, 30–35]. Furthermore, a scoping review of 70 studies from different settings around the world [5] reported high variability in burnout (25.12–74%) and stress (8.3–87.1%) prevalence among teachers. The variation in the prevalence of burnout reported by different studies may be related to variations in the period and location of data collection, measurement tool, heterogeneity in study designs, and partly to the variation in sample sizes [5].

The assessment of factors associated with burnout among our respondents showed that those who were younger, and those who had less than five years of teaching experience exhibited higher levels of burnout, which is consistent with findings from previous studies indicating the negative relationship between occupational burnout and younger age or limited teaching experiences [36, 37]. O’Brennan and colleagues [38] in the multilevel examination of burnout among school staff reported that teachers with long experience in the profession had lower levels of occupational burnout. The combination of being young and having less work experience may contribute to heightened burnout. In contrast, adult teachers, who have more experience and are generally more emotionally regulated, often possess better stress management skills [7]. Some research found that young teachers experienced more stress than their older colleagues, possibly due to feelings of failure, inadequacy, and undervaluation, which are more prevalent among ambitious and optimistic young teachers [39, 40].

The teaching profession is globally acknowledged as being predominantly a profession for women, which reflects the growing representation of women in this field [41]. However, the current study reveals a relatively low participation rate of female teachers, accounting for only 33%. Nevertheless, when compared to previous periods in Nepal, there has been a notable increase in the presence of women within the profession [42, 43]. Women may experience elevated stress levels than men due to additional commitments to family and childcare including the commitment to teaching and students. Our study’s findings support the existence of gender disparities in the severity of burnout, particularly regarding PE. This finding aligns with the prior research [44, 45], which affirms that burnout levels can vary based on gender, with women being more prone to reporting burnout than men. According to Taylor et al. [46], there are notable differences between genders when it comes to their biological, psychological, and behavioral reactions to stress. Nevertheless, it should be noted that mental health concerns are influenced by genetic factors, including gender. However, it is important to recognize that the stress response is not solely determined by genotype alone, as highlighted by Plomin et al. [47]. Similarly, some studies concluded that there were no significant gender differences in the overall prevalence of burnout among teachers [23, 48]. Beer & Beer [49] reported that both genders suffered burnout in similar ways.

Teachers who reported having poor health, inadequate physical fitness, minimal exercise, and sedentary leisure activities were more susceptible to experiencing burnout. Specifically, teachers with poor health conditions experienced higher levels of burnout. Several previous studies have argued that lifestyle factors play a crucial role in managing burnout [38, 45, 50, 51]. Our results indicate that promoting a healthy lifestyle among teachers can help to reduce their stress-related lifestyle outcomes. Therefore, it is expected that teachers need to recognize and effectively manage their stress by maintaining a balanced healthy work-life and avoiding negative outcomes [52]. Likewise, sleep patterns seemed to associate with particularly EE syndrome notably as reported by Gluschkoff and colleagues [53]. These findings were highlighted in previous studies suggesting that insufficient sleep [54] as well as mood fluctuations [55] contributes to burnout among teachers. It is plausible that sleep patterns and stress have a reciprocal relationship. Furthermore, the empirical evidence confirms the adverse effects of smoking on EE and PA.

Similarly, the teachers who consumed alcohol had higher levels of burnout syndromes than those who did not drink alcohol and it especially contributed to DP and PE syndromes. However, this finding should be cautiously considered as the assessment of alcohol consumption in this study was based on a general yes/no question and did not consider the quantity and frequency of consumption of alcohol. Teachers having some level of disability (e.g., hearing, visual, mobility-impaired) reported elevated stress in terms of EE than those without disabilities in our study. Similarly, we found that the teachers teaching special needs students had higher stress than those who were not involved in teaching students with special needs, particularly significant differences found in DP, which aligns with the findings of the previous studies [56–58] suggesting that working with inclusive students is linked to the development of DP. Teachers with poor work ability experienced higher levels of burnout compared to those with moderate and better work ability. Particularly, work ability was identified as one of the work-related variables associated with higher stress in all three sub-scales of MBI-ES with a strong association with DP and notable differences in EE and PA.

The current study possesses several strengths worth mentioning. Firstly, the random selection of sample clusters (schools) ensures that the findings can be generalized to a larger population of secondary school teachers in lower-middle-income countries. Secondly, the study participants exhibit homogeneity in terms of their social background, cultural practices, and geographical characteristics, which enhances the reliability and validity of the study results. We used the Maslach Burnout Inventory (MBI-ES) tool from Mind Garden (USA) which is widely recognized for its robustness and accuracy in measuring occupational burnout, especially in human services professionals. This tool has been extensively validated in previous studies [59, 60]. The significance of this study is further strengthened by the limited availability of similar research among the teachers of lower-middle-income countries like Nepal. Consequently, this study fills an important research gap by examining the levels of occupational burnout among teachers and their associations with demographic factors, lifestyle, health conditions, and work-related aspects.

The present study has some limitations to be considered. Firstly, the cross-sectional nature of the data restricts the possibility to establish causal relationships between occupational burnout and the factors investigated as potential determinants. The study relied on a self-reported web-based survey, which may have influenced participant responses and introduced a potential bias [61]. However, self-report measures may capture heterogeneity in responses from respondents regarding exposures rather than assuming an average level of exposure to any kind of phenomenon. Our findings are generalizable to secondary-level schoolteachers across Nepal with particular relevance for educators working in urban settings in lower-middle-income countries.

## Conclusion

The prevalence of occupational burnout among schoolteachers in Nepal was relatively high with the exhibition of moderate to high symptoms of EE 31%, DP 35%, and PA 27% of the representative sample. Poor work ability was the major contributor to the higher level of burnout and factors such as age, gender, poor health conditions, lack of physical fitness, disability conditions, sedentary behaviours, limited work experience, teaching students with special needs, smoking, alcohol consumption, and poor sleep quality were among the other contributors. Most of the contributing factors were modifiable, which indicates the need for interventions aimed at promoting a healthy lifestyle, enhancing work ability, and addressing work-related factors within this professional group. Nevertheless, effectively managing burnout requires the implementation of primary, secondary, and tertiary prevention strategies at both organizational and individual levels [62].

## Data Availability

The datasets generated during the current study are not publicly available due to ethic issues involving participant’s data and privacy but can be available from the corresponding author on reasonable request.
